# Supplementary material for: Modeling glioblastoma heterogeneity as a dynamic network of cell states
Source: Mol Syst Biol. 2021 Sep 16;17(9):e10105. doi: 10.15252/msb.202010105 (PMC8444284; doi:10.15252/msb.202010105)
Supplement: Supplementary file 6 — Source Data for Figure 5 [file MSB-17-e10105-s004.zip › Figure5A_sourcedata/GSEA_3071/hallmarks_stateII.GseaPreranked.1623742536692/HALLMARK_TNFA_SIGNALING_VIA_NFKB.html]

Details for gene set HALLMARK\_TNFA\_SIGNALING\_VIA\_NFKB[GSEA]

|  || Dataset | state23071 |
| Phenotype | NoPhenotypeAvailable |
| Upregulated in class | na\_pos |
| GeneSet | HALLMARK\_TNFA\_SIGNALING\_VIA\_NFKB |
| Enrichment Score (ES) | 0.20252758 |
| Normalized Enrichment Score (NES) | 1.1759483 |
| Nominal p-value | 0.22891566 |
| FDR q-value | 0.30910715 |
| FWER p-Value | 0.994 |
Table: GSEA Results Summary

  

Fig 1: Enrichment plot: HALLMARK\_TNFA\_SIGNALING\_VIA\_NFKB      
 Profile of the Running ES Score & Positions of GeneSet Members on the Rank Ordered List

  

| PROBE | GENE SYMBOL | GENE\_TITLE | RANK IN GENE LIST | RANK METRIC SCORE | RUNNING ES | CORE ENRICHMENT || 1 | EDN1 |  |  | 6 | 1.670 | 0.0319 | Yes |
| 2 | F3 |  |  | 13 | 1.555 | 0.0614 | Yes |
| 3 | LIF |  |  | 64 | 1.184 | 0.0726 | Yes |
| 4 | FOSL1 |  |  | 77 | 1.119 | 0.0920 | Yes |
| 5 | PTX3 |  |  | 139 | 0.981 | 0.0964 | Yes |
| 6 | GADD45B |  |  | 146 | 0.965 | 0.1142 | Yes |
| 7 | PLAUR |  |  | 185 | 0.893 | 0.1226 | Yes |
| 8 | INHBA |  |  | 196 | 0.876 | 0.1376 | Yes |
| 9 | DUSP5 |  |  | 274 | 0.781 | 0.1340 | Yes |
| 10 | SERPINE1 |  |  | 299 | 0.756 | 0.1431 | Yes |
| 11 | PHLDA2 |  |  | 332 | 0.726 | 0.1496 | Yes |
| 12 | KLF6 |  |  | 357 | 0.708 | 0.1578 | Yes |
| 13 | IL18 |  |  | 427 | 0.666 | 0.1539 | Yes |
| 14 | PDLIM5 |  |  | 526 | 0.613 | 0.1417 | Yes |
| 15 | BTG3 |  |  | 540 | 0.607 | 0.1506 | Yes |
| 16 | SPHK1 |  |  | 551 | 0.603 | 0.1602 | Yes |
| 17 | SPSB1 |  |  | 566 | 0.592 | 0.1685 | Yes |
| 18 | SDC4 |  |  | 583 | 0.585 | 0.1762 | Yes |
| 19 | DUSP1 |  |  | 599 | 0.578 | 0.1840 | Yes |
| 20 | KLF2 |  |  | 679 | 0.537 | 0.1751 | Yes |
| 21 | IER5 |  |  | 686 | 0.536 | 0.1843 | Yes |
| 22 | MYC |  |  | 790 | 0.502 | 0.1687 | Yes |
| 23 | GADD45A |  |  | 867 | 0.480 | 0.1593 | Yes |
| 24 | NFKB1 |  |  | 874 | 0.478 | 0.1674 | Yes |
| 25 | RHOB |  |  | 881 | 0.476 | 0.1754 | Yes |
| 26 | ETS2 |  |  | 923 | 0.466 | 0.1745 | Yes |
| 27 | ACKR3 |  |  | 962 | 0.454 | 0.1741 | Yes |
| 28 | HBEGF |  |  | 972 | 0.451 | 0.1808 | Yes |
| 29 | MAFF |  |  | 986 | 0.447 | 0.1865 | Yes |
| 30 | IER3 |  |  | 1010 | 0.442 | 0.1896 | Yes |
| 31 | GFPT2 |  |  | 1022 | 0.437 | 0.1956 | Yes |
| 32 | TNFAIP3 |  |  | 1070 | 0.424 | 0.1924 | Yes |
| 33 | CDKN1A |  |  | 1088 | 0.419 | 0.1965 | Yes |
| 34 | MAP2K3 |  |  | 1136 | 0.410 | 0.1930 | Yes |
| 35 | IRF1 |  |  | 1173 | 0.403 | 0.1921 | Yes |
| 36 | TNIP1 |  |  | 1184 | 0.399 | 0.1976 | Yes |
| 37 | TNFAIP8 |  |  | 1197 | 0.397 | 0.2025 | Yes |
| 38 | PPP1R15A |  |  | 1284 | 0.381 | 0.1887 | No |
| 39 | IL7R |  |  | 1360 | 0.366 | 0.1773 | No |
| 40 | EHD1 |  |  | 1413 | 0.354 | 0.1714 | No |
| 41 | IER2 |  |  | 1487 | 0.342 | 0.1601 | No |
| 42 | YRDC |  |  | 1492 | 0.342 | 0.1659 | No |
| 43 | CLCF1 |  |  | 1575 | 0.328 | 0.1520 | No |
| 44 | BIRC3 |  |  | 1626 | 0.320 | 0.1460 | No |
| 45 | KLF10 |  |  | 1662 | 0.315 | 0.1435 | No |
| 46 | JUN |  |  | 1725 | 0.307 | 0.1342 | No |
| 47 | CCND1 |  |  | 1753 | 0.304 | 0.1336 | No |
| 48 | SGK1 |  |  | 1806 | 0.298 | 0.1266 | No |
| 49 | CD83 |  |  | 1838 | 0.294 | 0.1247 | No |
| 50 | RIPK2 |  |  | 1892 | 0.286 | 0.1172 | No |
| 51 | NFIL3 |  |  | 2056 | 0.267 | 0.0820 | No |
| 52 | LDLR |  |  | 2128 | 0.260 | 0.0695 | No |
| 53 | EIF1 |  |  | 2139 | 0.258 | 0.0721 | No |
| 54 | DRAM1 |  |  | 2174 | 0.255 | 0.0687 | No |
| 55 | FOSL2 |  |  | 2216 | 0.250 | 0.0635 | No |
| 56 | PTPRE |  |  | 2265 | -0.255 | 0.0567 | No |
| 57 | ZBTB10 |  |  | 2459 | -0.279 | 0.0141 | No |
| 58 | KDM6B |  |  | 2691 | -0.314 | -0.0372 | No |
| 59 | CFLAR |  |  | 2781 | -0.332 | -0.0527 | No |
| 60 | PDE4B |  |  | 2788 | -0.333 | -0.0475 | No |
| 61 | SNN |  |  | 2873 | -0.349 | -0.0615 | No |
| 62 | CCNL1 |  |  | 2922 | -0.360 | -0.0663 | No |
| 63 | SQSTM1 |  |  | 3013 | -0.378 | -0.0812 | No |
| 64 | MARCKS |  |  | 3369 | -0.487 | -0.1599 | No |
| 65 | B4GALT5 |  |  | 3372 | -0.487 | -0.1507 | No |
| 66 | LAMB3 |  |  | 3384 | -0.491 | -0.1436 | No |
| 67 | NFE2L2 |  |  | 3389 | -0.493 | -0.1347 | No |
| 68 | PMEPA1 |  |  | 3390 | -0.494 | -0.1248 | No |
| 69 | VEGFA |  |  | 3532 | -0.560 | -0.1488 | No |
| 70 | BCL6 |  |  | 3542 | -0.566 | -0.1397 | No |
| 71 | G0S2 |  |  | 3545 | -0.568 | -0.1289 | No |
| 72 | TUBB2A |  |  | 3554 | -0.572 | -0.1194 | No |
| 73 | PHLDA1 |  |  | 3622 | -0.617 | -0.1238 | No |
| 74 | SAT1 |  |  | 3659 | -0.653 | -0.1197 | No |
| 75 | ID2 |  |  | 3664 | -0.654 | -0.1077 | No |
| 76 | LITAF |  |  | 3670 | -0.660 | -0.0957 | No |
| 77 | TGIF1 |  |  | 3728 | -0.707 | -0.0958 | No |
| 78 | DUSP4 |  |  | 3745 | -0.724 | -0.0853 | No |
| 79 | FJX1 |  |  | 3824 | -0.813 | -0.0885 | No |
| 80 | TSC22D1 |  |  | 3872 | -0.887 | -0.0825 | No |
| 81 | PLPP3 |  |  | 3900 | -0.946 | -0.0703 | No |
| 82 | FOS |  |  | 3911 | -0.966 | -0.0535 | No |
| 83 | BTG2 |  |  | 3917 | -0.999 | -0.0348 | No |
| 84 | NINJ1 |  |  | 3929 | -1.015 | -0.0173 | No |
| 85 | PNRC1 |  |  | 4022 | -1.397 | -0.0123 | No |
| 86 | TNC |  |  | 4039 | -1.549 | 0.0147 | No |
Table: GSEA details [plain text format]

  

Fig 2: HALLMARK\_TNFA\_SIGNALING\_VIA\_NFKB: Random ES distribution      
 Gene set null distribution of ES for **HALLMARK\_TNFA\_SIGNALING\_VIA\_NFKB**
